# Knockout of maternal *CD163* protects fetuses from infection with porcine reproductive and respiratory syndrome virus (PRRSV)

**DOI:** 10.1038/s41598-017-13794-2

**Published:** 2017-10-17

**Authors:** Randall S. Prather, Kevin D. Wells, Kristin M. Whitworth, Maureen A. Kerrigan, Melissa S. Samuel, Alan Mileham, Luca N. Popescu, Raymond R. R. Rowland

**Affiliations:** 1 Division of Animal Science, College of Food Agriculture and Natural Resources, University of Missouri, Columbia, MO 65211 USA; 2 Department of Diagnostic Medicine and Pathobiology, College of Veterinary Medicine, Kansas State University, Manhattan, KS 66506 USA; 3 Genus, plc, DeForest, Wisconsin USA

## Abstract

After infection of the porcine dam at about 90 days of gestation, porcine reproductive and respiratory syndrome virus (PRRSV) crosses the placenta and begins to infect fetuses. Outcomes of include abortion, fetal death and respiratory disease in newborn piglets. CD163 is the receptor for the virus. In this study, CD163-positive fetuses, recovered between 109 days of gestation or 20 days after maternal infection, were completely protected from PRRSV in dams possessing a complete knockout of the CD163 receptor. The results demonstrate a practical means to eliminate PRRSV-associated reproductive disease, a major source of economic hardship to agriculture.

## Introduction

Porcine reproductive and respiratory syndrome (PRRS) is the most economically important disease of swine in North America, Europe and Asia, costing North American producers approximately $600 million annually^1^. Losses are the result of respiratory disease in young pigs, poor growth performance, reproductive failure, and *in utero* infection^2^. The reproductive form of the disease accounts for an estimated 45% of losses, the result of abortions, dead fetuses, and respiratory disease in newborns. In its severest form, reproductive PRRS can result in 90% mortality of fetuses/neonates, along with increased mortality for the dams. Pigs that survive *in utero* infection become continuous sources of virus in downstream production phases, resulting in endemically infected herds^3^. The severest form of reproductive disease is associated with a group of highly virulent isolates referred to as atypical PRRSV^4,5^. Interestingly, many of the atypical PRRSV isolates emerged from PRRS-vaccinated farms^6^. In 2006, an atypical virus, called high pathogenic PRRSV (HP-PRRSV), appeared in China and continues to decimate pig populations in that country^7^. Since the standard commercial breeding facility contains about 5,000 sows, an outbreak of high mortality reproductive PRRS can have a devastating impact. To ensure sustainability of pork production and food security, solutions for the control of reproductive PRRS remain a priority. Vaccines have been unable to control the disease, largely because of genetic diversity within the structural proteins of the virus^8^. In practice, intensive biosecurity measures provide the only means of protecting the reproductive herd.

Along with lactate dehydrogenase-elevating virus (LDV) of mice, equine arteritis virus (EAV), and simian hemorrhagic fever virus (SHFV), PRRSV belongs to the family, *Arterviridae*. Structurally, the arteriviruses resemble togaviruses, but similar to coronaviruses, replicate via a nested 3′-co-terminal set of subgenomic mRNAs, which possess a common leader and a poly-A tail. Arteriviruses exhibit a tropism for macrophages and possess the capacity to establish subclinical persistent infections, as well as cause severe and fatal disease^9^.

The reproductive form of PRRS occurs following the infection of pregnant gilts or sows at about 90 days of the 114 day gestation period^10,11^. After an initial phase of replication in maternal macrophages, the virus crosses the placenta and begins to productively infect fetuses. The virus initially infects only a small number of fetuses, followed by horizontal transmission of virus from fetus to fetus^12^. The exact mechanism of how the virus crosses the placenta remains unknown, but could be similar to the infected “Trojan Horse” macrophage, previously described for LDV^13^. Unlike the alveolar macrophages in adult animals, the primary site of PRRSV replication in the fetus is the thymus^3^. Since the pig fetus becomes immunocompetent at about 70 days of gestation, PRRSV infection occurs in a fetal immune environment containing functional B and T cells^3,11^.

CD163 is a 130 kDa type 1 membrane protein composed of nine scavenger receptor cysteine-rich (SRCR) domains and two spacer domains along with a transmembrane domain and a short cytoplasmic tail. In addition to functioning as a virus receptor, CD163 exhibits several important functions related to maintaining normal homeostasis. For instance, following infection or tissue damage, CD163 functions as a scavenger molecule, removing haptoglobin-hemoglobin complexes from the blood^13^. The resulting heme degradation products regulate the associated inflammatory response^14^. CD163 as a receptor for PRRSV was first described by Calvert *et al*.^15^. Transfection of non-permissive cell lines with CD163 cDNA from a variety of species, including simian, human, canine, and mouse can make cells permissive for infection. We recently showed that pigs with a complete knockout (KO) of the *CD163* gene lack CD163 expression on macrophages and fail to support PRRSV infection^16,17^. Since *CD163* expression is a dominant trait and inherited in a classic Mendelian fashion, offspring possessing normal CD163 expression and function can be derived by crossing a KO *CD163* ^−/−^ female pig with a wildtype (WT) *CD163* ^+/+^ male. For this study, *CD163* KO gilts were bred with WT boars producing heterozygous, *CD163* ^+/−^ fetuses. The hypothesis to be tested was that the presence of the CD163 KO genotype of the dam would be sufficient to protect fetuses following maternal infection with PRRSV.

## Results and Discussion

A detailed description of the knockout alleles used in this study is shown in Table 1. Each knockout allele possessed a mutation in exon 7 that was predicted to result in a codon frameshift followed by a premature stop codon in the mRNA. The matings between WT and *CD163* KO parents are summarized in Table 2. The first group of three dams, which served as positive infection controls, were *CD163* ^+/+^ dams carrying *CD163* ^+/+^ fetuses (++/++ group). A second group (−/+−) were *CD163* ^−/−^ dams carrying *CD163* ^+/−^ fetuses. In this group, the *CD163* ^−/−^ dams are unable to support PRRS replication, while the *CD163* ^+/−^ fetuses retain susceptibility to PRRS infection. And finally, a third group (−/−) consisted of *CD163* ^−/−^ dams carrying *CD163* ^−/−^ fetuses. For the last group, both dams and fetuses should be resistant to infection.

**Table 1:** *CD163* alleles used in this study.

Allele	Description
A	Wild Type
B	Knockout (7 bp insertion in exon 7)
C	Knockout (2 bp insertion in exon 7)
D	Knockout (11 bp deletion in exon 7
E	Knockout (1382 bp deletion that included part of exon 7 and 8 with an 11 bp insertion in exon 7)

**Table 2:** *CD163* parental and fetal genotypes used in this study.

Gilt No.	*CD163* Genotype			Collection of Fetuses		
	Parents*^1^		Fetus	Day of	Day of	No. of
	Male	Dam		Infection*^1^	Gestation*^2^	Fetuses
138	A/A	A/A	+/+	91	106	16
139*^3^	A/A	A/A	+/+	91	106	14
140	A/A	A/A	+/+	91	106	12
84	A/A	B/C	+/−	89	109	14
87	A/A	B/C	+/−	89	109	17
122	A/A	E/C	+/−	89	109	11
86	C/D	B/D	−/−	90	109	7
121	C/D	E/D	−/−	90	109	9

*^1^ *CD163* alleles are identified in Table 1.

*^2^Gestation day when dams were infected.

*^3^Gestation day when fetuses were removed.

*^4^PRRSV-infected dam aborted at 106 days of gestation.

**Figure 1:**
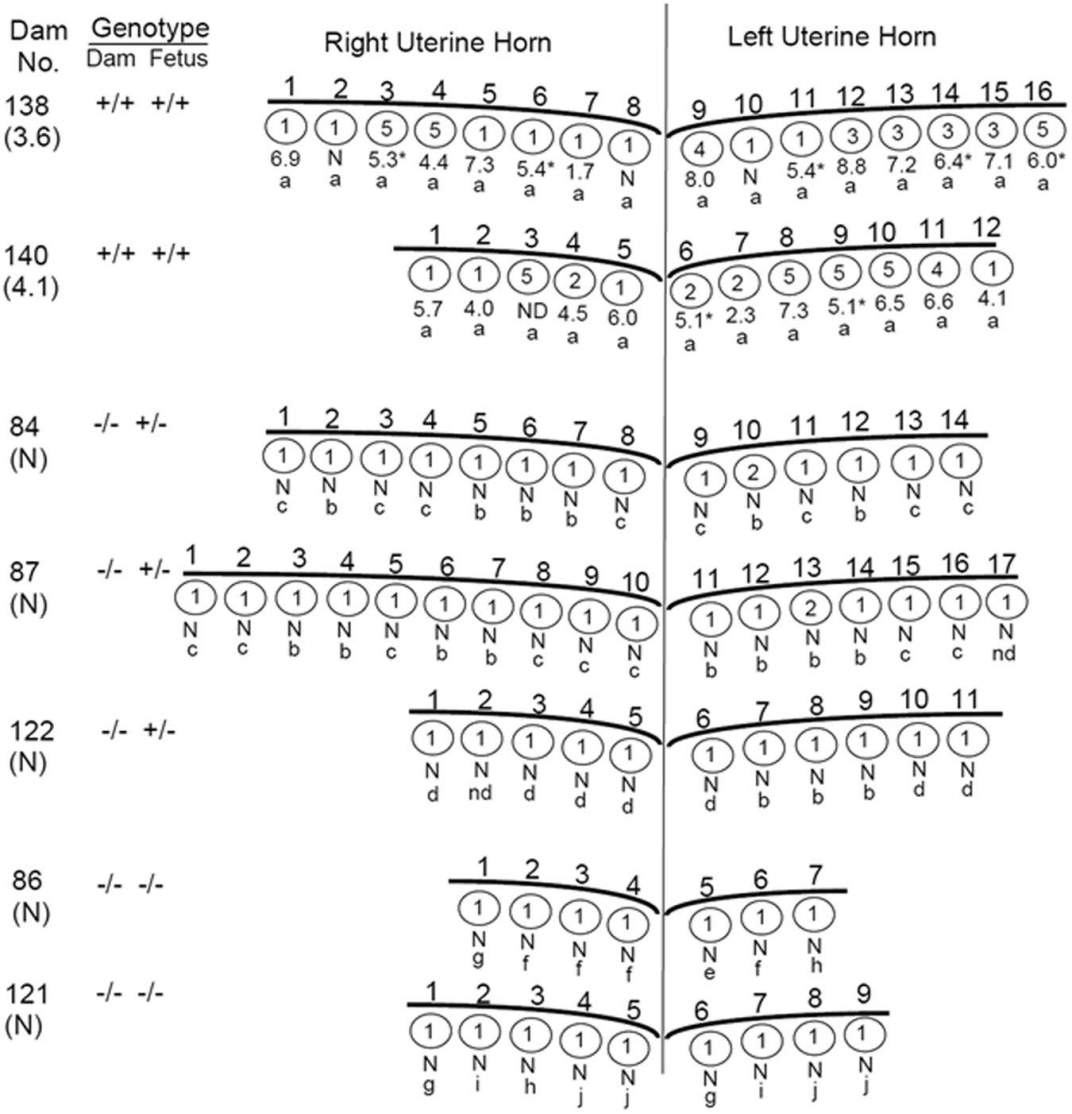
Outcomes following maternal infection with PRRSV. The numbers on the left identify each dam (see Table 1). Below each dam in parentheses is the result for PRRS PCR in serum, measured as log_10_ templates per reaction. “N” is negative for PRRSV nucleic acid (Ct > 39). Fetuses are identified by number and relative position within each uterine horn. Asterisks identify fetal PCR samples obtained from abdominal fluid. The number below each fetus is the result for PRRS PCR in fetal serum (log_10_ templates per reaction). The number within each circle refers to the presence of anatomical pathology: 1) normal fetus; 2) small fetus; 3) placental changes, such as detached placenta and/or necrosis; 4) meconium stained fetus; 5) fetus is dead and necrotic. Lower case letters identify the genotype of the individual fetuses (see Table 1). Key: a, A/A; b, C/A; c, B/A; d, E/A; e, B/C; f, B/D; g, D/C; h, D/D; i, E/C; j, E/D; ND not determined because the fetus was necrotic; nd, genotype was not determined.

Clinical signs in the infected WT dams included lethargy and transient inappetance. The KO dams showed no clinical signs. During the study period, one WT dam, No. 139, aborted on day 106 of gestation (15 dpi). PRRSV nucleic acid, measured at 7 dpi, showed a viremia level for Dam No. 139 of 5.5 log_10_ templates per reaction, demonstrating the presence of a productive PRRSV infection. Between 15 and 20 dpi, all remaining dams were euthanized and uterine horns immediately removed. Beginning at the tip of each horn, fetuses and placentas were removed, assessed for the presence of anatomic pathology. A blood sample was obtained from each fetus. If blood was not obtainable, a sample of fluid was collected from the abdominal cavity. The number of fetuses recovered from each dam is listed in Table 2. For the *CD163* WT group (++/++) (including the dam that aborted), the number of fetuses were 16, 14 and 12 (mean = 14.0). The *CD163* KO dams carrying the *CD163* ^+/−^ fetuses (−/+− group) yielded 14, 16 and 11 fetuses (mean = 13.6). For the *CD163* KO dams carrying *CD163* KO fetuses (−−/−−group), the numbers of fetuses were 7 and 9. The results for fetal viremia and gross pathology are summarized in Fig. 1 and Table 3. At the anatomic level, 50% and 72% of fetuses derived from the two *CD163* WT (++/++) dams, No. 138 and No. 140, showed some degree of pathology, including smaller than normal fetuses (11% of all fetuses), fetuses with detached or necrotic placentas (14%), meconium staining (7%), and fetuses that were dead and necrotic (25%). The pathology observations are typical of reproductive PRRS. The same litters showed a high rate of PRRSV infection, with 92% of the fetuses testing positive for the presence of PRRSV nucleic acid. The PCR results for the fetuses from the WT dams illustrate two important properties of fetal PRRSV infection. First, there was a wide variation between fetuses in the concentration of virus detected in serum, the result of fetuses becoming infected at different times. Secondly, the level of viremia was not always correlated with pathology. For example, Fetus No. 5 from Dam No. 138 possessed a high level of viremia (7.3 log_10_ templates per reaction) and yet the fetus appeared unaffected. The reason for the discrepancy between viremia and the pathology is unclear. One possibility is that fetal pathology is the result of tissue damage that occurs on the maternal side and not related to the level of fetal viremia. In the field, these normal, but infected newborn piglets can function as “supershedders”, which facilitate the rapid dissemination of PRRSV throughout a production system. For the −−/+− group (dams No. 84, 87 and 122), all fetuses appeared normal, with the minor exception of two fetuses that were smaller than the other littermates. The smaller than normal size is likely a consequence of crowding within the uterine horn that decreases the surface area of the placenta, thus restricting the growth of the developing fetus. All dams and fetuses in the −−/+− group were negative for the presence of PRRSV nucleic acid. For the last group, −–/−−, there was no visible pathology, and all dams (No. 86 and 121) and fetuses were negative for PRRSV nucleic acid.

**Table 3:** Summary of fetal infection and pathology*^1^.

Gilt No.	Dam Genotype	Fetus Genotype	Total No. Fetuses	Dam Viremia*^2^	No. Infected Fetuses	Pathology*^3^
138	+/+	+/+	16	3.6	13 (80%)	8 (50%)
139*^4^	+/+	+/+	14	5.5	ND	ND
140	+/+	+/+	12	4.1	11 (92)	8 (72)
84	−/−	+/−	14	N	0 (0)	1 (07)
87	−/−	+/−	17	N	0 (0)	1 (06)
122	−/−	+/−	11	N	0 (0)	0 (0)
86	−/−	−/−	7	N	0 (0)	0 (0)
121	−/−	−/−	9	N	0 (0)	0 (0)

*^1^The table is a combined summary of the data presented in Table 2 and Fig. 1.

*^2^Viremia shown as log10 virus nucleic templates per PCR.

*^3^Fetuses showing pathology as described in the legend in Fig. 1.

*^4^Gilt aborted prior to recovery of fetuses.

The results from this study clearly demonstrate that the absence of CD163 in the dam is sufficient to protect the PRRSV-susceptible fetus. Although CD163-positive offspring derived from *CD163* KO dams are susceptible to virus immediately after birth, the protection from PRRSV *in utero* provides a means to eliminate a major source of economic loss and animal suffering.

## Methods

### *CD163* gene modification

The CRISPR/Cas9 methods used to generate all of the KO alleles are described in detail in Whitworth *et al*.^18^. The specific edits for alleles A, B, D and E are described in Whitworth *et al*.^18^. The specific edit in Allele C (2 bp insertion) is described in Whitworth *et al*.^16^. The alleles, described in Table 1 were identified based on DNA sequencing. The knockout genotype was confirmed by the absence of CD163 expression, which was measured by staining alveolar macrophages with anti-CD163 mAb, 2A10, as described in Wells *et al*.^17^

### PRRSV infection

The PRRSV strain used in this study, NVSL 97–7895 (NVSL), is a laboratory strain isolated in 1997 from a herd in Southeast Iowa, USA that was experiencing a PRRS abortion storm^4^. The virus, maintained as a low passage isolate, was propagated and titered on MARC-145 cells. At 89 to 91 days of gestation, gilts were inoculated with 10^5^ TCID_50_ of virus diluted in 5 ml of culture medium. One half of the inoculum was administered by intramuscular injection and the remainder administered intranasally. All gilts were maintained in an environment that allowed for the continuous exposure to virus shed by infected pen mates. Blood samples were taken from the gilts prior to infection, 7 days post-inoculation (dpi), and at the time of euthanasia. PRRSV nucleic acid was measured by isolation of total RNA from serum followed by reverse transcriptase real-time PRRSV PCR (Tetracore, Rockville, MD). A standard curve was generated using the quantification standards supplied in the RT-PCR kit. Results were reported as log_10_ templates per 25 µl reaction, which approximates the number of viral RNA templates per ml of blood.

### Ethics statement

Experiments involving animals and virus were performed in accordance with the Federation of Animal Science Societies Guide for the Care and Use of Agricultural Animals in Research and Teaching, the USDA Animal Welfare Act and Animal Welfare Regulations, or according to the National Institutes of Health’s Guide for the Care and Use of Laboratory Animals, and were approved by the Kansas State University and University of Missouri institutional animal care and use committees and institutional biosafety committees. Animals were humanely euthanized by pentobarbital overdose following the American Veterinary Medical Association (AVMA) guidelines for the euthanasia of animals, and all efforts were made to minimize suffering.
